# Applicants' success in the ethics entrance exam: A cross-sectional study

**DOI:** 10.1177/09697330231204999

**Published:** 2023-10-30

**Authors:** Jonna Vierula, Tiina Karihtala, Niina Ervaala, Kati Naamanka, Elina Haavisto, Kirsi Talman

**Affiliations:** 3254 Laurea University of Applied Sciences; 52907 Metropolia University of Applied Sciences; 11325 South-Eastern Finland University of Applied Sciences; 8056 Turku University of Applied Sciences; 7840 Tampere University; 52907 Metropolia University of Applied Sciences

**Keywords:** Ethical competence, ethical awareness, higher education, social and healthcare, student selection, values-based recruitment

## Abstract

**Background:**

Student selection is the first step in recruiting future social and healthcare professionals. Ethically competent professionals are needed in social and healthcare. It is important to select applicants who have the best possible abilities to develop their ethical competence in the future. Values-based recruitment has been used to inform the recruitment and selection of higher education applicants. However, objective and valid tests in student selection are needed.

**Aim:**

To assess social and healthcare applicants’ success and related factors in the ethics section of the universities of applied sciences digital entrance examination (UAS Exam) to undergraduate degree programmes.

**Research design:**

A cross-sectional design was used.

**Participants and research context:**

Social and healthcare applicants needed to identify ethical situations in the ethics section of a national digital entrance examination (UAS Exam) in autumn 2019 (between 29 October and 1 November) in 20 Finnish universities of applied sciences.

**Ethical considerations:**

The process for the responsible conduct of research was followed in the study. Ethics committee approval was obtained from the Human Sciences Ethics Committee in the Satakunta region (27 September 2019). Approval to undertake the study was obtained from the participating universities of applied sciences. Participation to the study was voluntary and based on informed consent.

**Results:**

The applicants’ (*n* = 8971) mean scores were 7.1/20 (standard deviation 6.5), and 22.7% of the applicants failed the ethics section. Age, previous education, and place of birth (own/parent) explained the applicants’ success in the ethics section (total score and failed exam results).

**Conclusion(s):**

Applicants’ success in the ethics section varied indicating that future students may have a different basis to develop their ethical competence. This may impact on (new) students’ learning, especially in practical studies.

## Introduction

To provide high-quality care, ethically competent professionals are needed in the field of social and healthcare.^1,2^ Ethically competent professionals understand the importance of ethics, can empathise with another person’s perspective, follow ethical principles, and address ethical issues in their daily work.^3^ Furthermore, (future) professionals need to be able to identify, examine, assess, and make decisions about ethical issues.^4^ Higher education student selection is the first step in recruiting future professionals. Therefore, higher education institutions offering social and healthcare programmes need to select students who have the necessary abilities to succeed in their studies and in their profession.^5^ Moreover, the assessment in the student selection phase should focus on factors reflecting the requirements of professional education.^6–8^

Recently, values-based recruitment (VBR) has been used to inform the recruitment and selection of individuals, such as higher education applicants; this is done based on their ability to demonstrate the values required for their role as a healthcare professional.^9^ It has been stated that VBR can ensure that students have the potential to become ethically competent professionals.^10^ Furthermore, the importance of assessing ethical factors, such as ethical values and action within healthcare student selection has been identified.^11^ The aim of social and healthcare programmes is to educate competent professionals. It is a common interest for educational institutions and working life that programmes select students with the best possible abilities to develop their ethical competence as social and healthcare students encounter ethical situations at an early stage in their studies in practice placements. However, applicants are not expected to possess ethical competence yet, since ethical competence develops in education during theoretical and practical studies and in working life, along with life and work experience, being constantly evolving. Furthermore, objective and valid tests in student selection are needed. Therefore, in this study, we assessed objectively one dimension of ethical awareness, specifically social and healthcare applicants’ ability to identify ethical situations.

## Background

Ethical competence can be considered a broad umbrella concept that facilitates other professional competence areas.^2,12^ Ethical competence can be defined as the conscious decisions made within a given situation, acting responsibly and considering the legal standards and economic, ecological, and social consequences.^13^ Ethical competence comprises, at least, ethical sensitivity, awareness to identify ethical situations, character strength, ethical knowledge, ethical reflection, judgement skills/ethical decision-making, ethical action, and ethical behaviour.^2,14^ According to Norman (1991),^15^ the first dimension of ethical competence includes the knowledge needed to identify ethical issues; the second dimension includes the analysis of the conflict and sensitivity towards competing values/principles; and the third dimension ensures that these decisions are actualised. To act ethically competently, professionals must first recognise that an ethical situation exists before any action can occur.^16,17^ Furthermore, healthcare professionals must first recognise the potential ethical repercussions of their actions to be able to solve problems and take the patient/client needs under consideration.^16,18^ This can be referred as ethical awareness. Ethical awareness involves the identification of the ethical content of the situation, namely, the ability to identify ethical content/problems and their possible consequences/implications.^18–20^ Moreover, ethical awareness includes the awareness of professionals’ roles and responsibilities in a given situation.^19,21,22^ Awareness of the ethical nature of practice is a component of ethical sensitivity, which has been identified as a component of ethical decision-making and thus part of ethical competence.^16,20^

VBR in social and healthcare has been discussed both in the contexts of student selection and clinical practice;^9,10^ the aim is to select individuals based on their ability to establish the values required as a healthcare professional,^9^ such as compassion, benevolence, respect, and preserving the dignity of the patient.^10^ In the student selection context, VBR methods are mainly based on different kinds of interviews, such as multiple mini-interviews (MMIs),^23,24^ personal statements or group activities.^25^ In medical student selection, the Situational Judgement Test has been used to assess applicants’ integrity (e.g. Husbands et al.,^26^). According to Patterson et al.,^10^ structured methods (e.g. situational judgement tests, structured interviews and MMIs) are considered appropriate for VBR, whereas unstructured methods (including personal statements or other subjective assessments) are seen as inappropriate. Altogether, measuring values and ethical competence is challenging because values and ethical competence develop throughout life and are affected by personality, social interaction, culture, and role models (e.g. Patterson et al.,^10^). VBR has been criticised for lacking clarity regarding what is being measured and having limited critical evaluation because the values are subjective to interpretation.^27^ VBR is also criticised for basing recruitment on individual values and behaviours^28^ which may not be relevant attributes in student selection context. Furthermore, VBR seems to lack the discriminatory power in terms of selecting people with right values or rejecting people who do not demonstrate the required values.^29^ Therefore, more evidence is required to make sure that selection decisions are based on best practice.^24,28^ For this, an objective and valid test in student selection is needed.

Internationally, student selection practices vary within and between higher education institutions.^30^ In many countries, including Finland, selective admission processes are used to rank students within the selection process, where selective admission means that there are more applicants than study spots available; this highlights the importance of objective and fair selection methods.^31,32^ In Finland, student selection practices were harmonised and developed in the higher education sector during the years 2017–2020, and as a result, the Finnish national digital universities of applied sciences entrance examination (UAS Exam) was established in autumn 2019.^33^ The UAS Exam assesses bachelor (undergraduate) degree applicants’ general readiness for higher education studies and includes several exam sections. Applicants will need to achieve a pass score from each section and the final selection decision is based on the total score of the exam. As part of the UAS Exam, the ethics section, which is a structured and objective assessment method for the applicants of social and healthcare programmes, was developed. The structure and content of the ethics section were determined using a mixed methods and instrument design.^34^ The content was identified based on a literature review and document analysis.^35^ Based on the results of a literature review, the first version of the ethics section measured the applicants’ ability to identify ethical situations. Ethical situations were based on the shared values of social and healthcare (e.g. respect for life, autonomy, humanity, and privacy) identified through the document analysis of ethical guidelines and principles. However, previous professional knowledge in social and healthcare was not required from the applicants to perform the ethics section.

The purpose of the current study was to assess social and healthcare applicants’ success and related factors in the ethics section of the universities of applied sciences digital entrance examination (UAS Exam) to undergraduate degree programmes. The ultimate goal was to develop student selection practices to select applicants able to develop their ethical competence during their studies and in their future professions. This study was part of a wider research project to develop universities of applied sciences student selection.

The research questions of this study were:

(1) How do undergraduate social and healthcare applicants succeed in the ethics section of the UAS Exam?
(2) What factors are related to undergraduate social and healthcare applicants’ success in the ethics section of the UAS Exam?

## Methods

### Study design

The present study was cross-sectional in design, utilising the results of the ethics section of the digital UAS Exam and demographic data from the digital exam system.

### Setting and participants

Altogether, 20 universities of applied sciences participated and used the UAS Exam between 29 October and 1 November 2019 to select their students. The exam was organised in 27 geographically spread-out locations. In each location, the exam was implemented under supervision with a closed network environment. The applicants used their own devices to access and complete the digital exam and were informed about the study before the exam on the official UAS Exam website. The information sheet was presented to the applicants again in the digital exam system before the informed consent was asked and before the applicants started the exam.

All the undergraduate (bachelor level) applicants to the study fields of social and healthcare participating in the UAS Exam (*N* = 11,720) were eligible to participate, and all consenting participants (*n* = 8972, response rate 76.6%) were included (Table 1). The study participants were mostly applicants whose first study choice (out of six possible) was in social or healthcare programmes (*n* = 8561, 95.4%). (Table 1)

**Table 1.** Participants (*n* = 8972) and their study programmes based on their first study choice.

Study programme	f	%
Community education	80	0.89
Elderly care (social services)	38	0.42
Social services	3351	37.35
Biomedical laboratory technology	170	1.89
Emergency nursing	708	7.89
Physiotherapy	617	6.88
Midwifery	275	3.07
Optometry	97	1.08
Radiography	101	1.13
Nursing	1957	21.81
Dental hygiene	107	1.19
Public health nursing	766	8.54
Occupational therapy	253	2.82
Sports coaching and management	41	0.46
**TOTAL**	**8561**	**95.43**
Other	411	4.58
**TOTAL**	**8972**	**100.00**

Note. Other (*n* = 411) describes the applicants who chose one of the study programmes above as their second, third, fourth, fifth, or sixth priority (in Finland, higher education applicants can apply altogether six study programmes with one application).

### Instrument

The ethics section of the UAS Exam assessed the applicants’ ability to identify ethical situations. The section included three scenarios (Table 2). Each scenario included a description of everyday life situations, followed by 10 statements with dichotomous answer options (true/false). For example, a situation describing fellow students’ discussion about plagiarism was presented and followed by 10 statements where the applicant needed to identify if each included ethical aspects or not (e.g. honesty). Correct answers yielded 0.5 or 1 points, and wrong answers yielded penalty scores of −0.25 or −0.33, respectively (Table 2). The penalty scores were used to avoid guessing behaviour. The minimum pass score was set to 1 point. The total score range of the ethics section was −8.3–20 points, comprising added responses from each question, hence resulting in sum scores (Table 2). Unfortunately, the more specific presentation of the concrete exam questions is not possible in this paper due to the use of exam questions in a high-stakes test.

**Table 2.** Structure of the ethics section of the UAS exam in autumn 2019.

Content of assessment	Questions	Scoring	Timing
Ability to identify ethical situations	Scenario 1: 10 true/false statements	0.5 pt/correct answer, −0.25 pt/wrong answer, 0 pt/don’t know	30 min
		**TOTAL: 5 pt**	
	Scenario 2: 10 true/false statements	0.5pt/correct answer, −0.25 pt/wrong answer, 0 pt/don’t know	
		**TOTAL: 5 pt**	
	Scenario 3^a^: 10 true/false statements	1 pt/correct answer, −0.33 pt/wrong answer, 0 pt/don’t know	
		**TOTAL: 10 pt**	
		**TOTAL: 20 pt**	
		Minimum passing score +1 pt^b^	

^a^The scoring of the third scenario differed from other scenarios. The third scenario included a longer description of everyday life situations. In addition, the maximum points of the ethics section needed to be 20 pt.

^b^The number of points (pt) an applicant needed to acquire to be applicable for selection.

The validity of the ethics section of the UAS Exam has been evaluated as part of the larger research project where the validity of all the UAS Exam sections were analysed.^33,35^ A digital Delphi survey design aiming for consensus with 10 experts (response rates 90% and 70% for the two rounds) was used to confirm the content validity of the ethics section exam questions. The experts evaluated clarity and difficulty level of the questions, accuracy of the correct answer, and time usage. The evaluated and modified questions were pilot tested with social and healthcare applicants (*n* = 1288). An item-level analysis with descriptive statistics and item response theory was used to evaluate the functionality and quality of the test questions, such as item parameters (difficulty, discrimination, pseudoguessing) and distractor analysis. Based on the results of the pilot testing, the difficulty level of the questions varied from rather easy to rather difficult (on a scale of easy, rather easy, rather difficult, difficult), and some questions were modified to decrease the difficulty level.^33,35^

### Data collection

Success in the ethics section was measured using exam results, namely, total score of the ethics section and failed exam results. If the applicant scored below the minimum pass score, the ethics section failed. Total scores were automatically calculated, and the exam results were applicable from the digital exam system. Also, background information (age, gender, previous education, socioeconomic background, place of birth [own, parents’], study field) was collected from the digital exam system.

### Data analysis

Statistical analysis was undertaken with the support of a qualified statistician using the Statistical Analysis Software (SAS 9.4®).^36^ Background information was applicable from 8972 applicants, but exam results could only be obtained from 8971 applicants; thus, one value was missing. Frequencies, percentages and measures of central tendency were calculated to describe the applicants’ exam results and demographic characteristics. Tukey’s test in post hoc multiple group comparisons was used to examine the factors related to applicants’ success (total score) in the ethics section of the UAS Exam. Logistic regression analysis was used to explain the applicants’ failed exam results and related factors. An analysis of the applicants’ failed exam results and related factors (logistic regression) was performed to support the previous analysis of examining the factors related to applicants’ success. This was done because it was considered important to analyse if similar background factors related to high scores (success) explain the very poorest scores (i.e. the failed result). For statistical analyses, the level of statistical significance was set at 0.05. Analysis was conducted as part of wider analyses focussing on the exam results in all the UAS Exam sections, in which the background variable ‘study field’ was included. However, these results are not reported because only students applying to the social and healthcare field undertook the ethics section of the exam.

### Ethical considerations

The process for the responsible conduct of research was followed.^37,38^ Ethics committee approval was obtained from the Human Sciences Ethics Committee in the Satakunta region (27 September 2019). Approval to undertake the study was obtained from the participating universities of applied sciences. An information sheet was provided to the participants. Participation was voluntary and based on informed consent, which was obtained from the participants in the digital exam system before the applicants started the exam. Confidentiality and anonymity were protected.

## Results

### Applicants’ demographic characteristics

Most applicants (38.09%, *n* = 3417) were over 29 years old and female (84.83%, *n* = 7611) (Table 3) representing typical Finnish social and healthcare applicants’ characteristics. In Finland, it is typical that many UAS applicants do not enter higher education directly from upper secondary school. In addition, social and healthcare education in Finland is female-dominated. The applicants were mainly high school graduates (37.84%, *n* = 3395) or had a vocational diploma (36.45%, *n* = 3270). Most reported that their parents were manual workers (socioeconomic background). Less than 10% (*n* = 695) of the applicants were born outside Finland. Here, 11.46% (*n* = 1028) of the applicants reported that at least one of their parents was born outside of Finland (Table 3).

**Table 3.** Demographic factors of the participants (*n* = 8972).

Demographic factor		f	%
Age			
<20		838	9.34
20–24		3041	33.89
25–29		1676	18.68
>29		3417	38.09
**Gender**			
Male		1361	15.17
Female		7611	84.83
**Previous education**			
High school		3395	37.84
Vocational school		3270	36.45
Double qualification (high school and vocational school)		241	2.69
Higher education degree		914	10.19
Other^a^		1152	12.84
**Socio-economic background (father)** ^b^			
Self-employed persons		1555	17.33
Upper-level employees (with administrative, managerial, professional and related occupations)		1291	14.39
Lower-level employees (with administrative and clerical occupations)		1437	16.02
Manual workers		3291	36.68
Students		138	1.54
Pensioners		883	9.84
Others (unemployed)		377	4.20
**Socio-economic background (mother)** ^b^			
Self-employed persons		721	8.04
Upper-level employees (with administrative, managerial, professional, and related occupations)		912	10.16
Lower-level employees (with administrative and clerical occupations)		2128	23.72
Manual workers		3790	42.24
Students		164	1.83
Pensioners		795	8.86
Others (unemployed)		462	5.15
**Place of birth (own):** Born in Finland			
Yes		8277	92.25
No		695	7.75
**Place of birth (parent):** One parent or both parents born outside Finland			
Yes		1028	11.46
No		7944	88.54

^a^‘Other’ refers to a specialised vocational degree or other than Finnish secondary or tertiary education, such as international matriculation examination or international higher education degree.

^b^Categorisation of the socio-economic groups is based on the classification provided by the Statistics Finland.

### Applicants’ success in the ethics section of the exam

**Figure 1.**
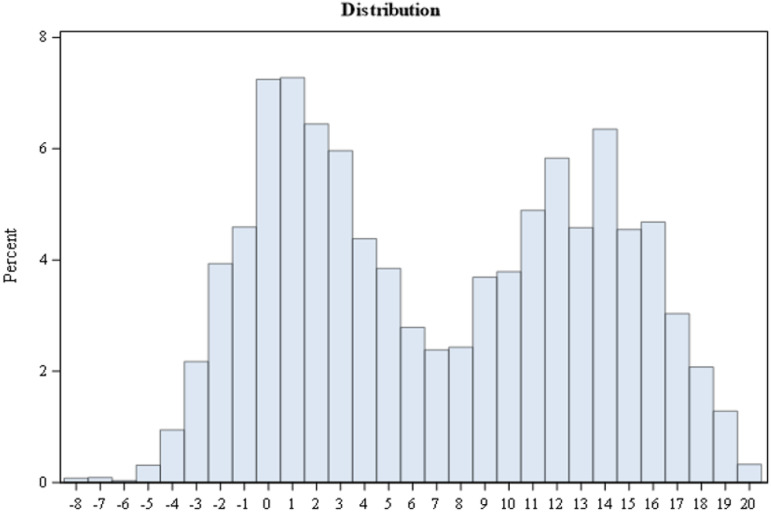
Distribution of participants’ total scores in the ethics section.

In the ethics section, the applicants’ (*n* = 8971) mean scores were 7.1 (standard deviation 6.5), the range (min-max.) was -8.30–20 points, and the median was 6.4. The mean scores (7.1) were below the centre of the positive score range (10 points) indicating that the ethics section was somewhat difficult for the applicants. Less than half of the applicants (39.7%, *n* = 3561) scored above the centre of the positive score range (10 points). Based on the standard deviation (6.5) of the total scores, the applicants’ performance in the ethics section varied, and the test (exam section) was able to rank order the applicants. The distribution of the applicants’ total scores is demonstrated in Figure 1. Out of the 8971 applicants, 22.7% (*n* = 2036) received a failed exam result (scored less than the minimum pass score +1 point) and, thus, succeeded poorly in the ethics section.

Tukey’s test in post hoc multiple group comparisons was used to examine the factors related to applicants’ success (total score) in the ethics section of the UAS Exam. As a result, age, previous education, and place of birth (own/parent) were statistically significantly related to applicants’ success (total score) in the ethics section. (Table 4). Older applicants scored better than younger applicants. High school graduates and applicants with previous higher education degrees scored better than those with a vocational diploma, double qualification (i.e. the applicant has completed both an upper secondary vocational qualification and the matriculation examination), or other previous education. Applicants who were born or who had one or both parents born in Finland scored better than applicants who were born or who had one or both parents born outside Finland (Table 4).

**Table 4.** Factors related to participants’ (*n* = 8971) success (total score) in the ethics section of the UAS exam.

Background variables	Difference between means	95% confidence interval	*p*-value
Age			
20–24 vs. > 29	−3.31	−3.72 – −2.90	<.0001
25–29 vs. > 29	−2.03	−2.49 – −1.57	<.0001
20–24 vs. 25–29	−1.28	−1.75 – −0.81	<.0001
<20 vs. 25–29	−2.06	−2.71 – −1.40	<.0001
<20 vs. 20–24	−0.77	−1.37 – −0.18	0.0047
<20 vs. > 29	−4.08	−4.70 – −3.47	<.0001
**Previous education**			
Vocational school vs. higher education degree	−4.04	−4.66 – −3.43	<.0001
Vocational school vs. high school	−3.20	−3.61 – −2.80	<.0001
Vocational school vs. double qualification^a^	−1.90	−3.00 – −0.82	<.0001
Higher education degree vs. other^b^	4.82	4.10 – 5.56	<.0001
Higher education degree vs. high school	0.84	0.21 – 1.48	0.0029
Vocational school vs. other^b^	0.78	0.22 – 1.34	0.0013
Double qualification^a^ vs. high school	−1.30	−2.38 – −0.23	0.0086
Double qualification^a^ vs. other^b^	2.68	1.53 – 3.83	<.0001
Other^b^ vs. high school	−4.00	−4.55 – −3.41	<.0001
Double qualification^a^ vs. higher education degree	−2.14	−3.33 – −0.96	<.0001
**Place of birth/own**			
Born in Finland vs. born outside Finland	2.46	1.85 – 3.08	<.0001
**Place of birth/parent**			
One parent/both parents born outside Finland vs. one parent/both parents born in Finland	−1.33	−1.84 – −0.82	<.0001

Note. Analysis is based on Tukey’s test in post hoc multiple group comparisons. Only the statistically significantly related background variables are included in the table.

^a^Double qualification = high school and vocational school.

^b^‘Other’ refers to a specialised vocational degree or other than Finnish secondary or tertiary education, such as international matriculation examination or international higher education degree.

Logistic regression analysis was used to explain the applicant’s failed exam results and related factors. As a result, the same background variables (age, previous education, place of birth [own/parent]) that were related to participants’ success (total score) in the ethics section, were also statistically significantly related to applicants’ failed exam results (Table 5). Younger applicants were more likely to have a failed exam result compared with applicants >29 years old. Applicants with vocational diplomas and other educations were more likely to have failed exam results compared with high school graduates and applicants with higher education degrees. Applicants who were born or who had one or both parents born outside Finland were more likely to have failed exam results compared with applicants who were born or who had one or both parents born in Finland (Table 5).

**Table 5.** Background variables explaining participants’ failed exam results in the ethics section of the UAS exam (*n* = 2036/*N* = 8971).

Background variables	OR	95% confidence interval	*p*-value
Age			
<20 vs. >29	2.57	2.13 – 3.09	<.0001
20–24 vs. >29	2.11	1.85 – 2.41	<.0001
25–29 vs. >29	1.73	1.49 – 2.00	<.0001
**Previous education**			
Higher education degree vs. vocational school	0.45	0.36 – 0.56	<.0001
Other^a^ vs. vocational school	1.17	1.00 – 1.36	0.0470
High school vs. vocational school	0.59	0.52 – 0.66	<.0001
Double qualification^b^ vs. vocational school	0.67	0.49 – 0.92	0.0134
**Place of birth/own**			
Born outside Finland vs. born in Finland	1.77	1.41 – 2.23	<.0001
**Place of birth/parent**			
One parent/both parents born in Finland vs. one parent/both parents born outside Finland	0.76	0.62 – 0.92	0.0050

Note. Analysis is based on logistic regression analysis. Only the statistically significantly related background variables are included in the table. OR: Odds Ratio. In the analysis, the group on the left is compared to the group on the right. When the result (OR) is more than 1, there are more failed exam results in the left group than in the right one. When the result (OR) is less than 1, there are fewer failed exam results in the left group than in the right one.

^a^‘Other’ refers to a specialised vocational degree or other than Finnish secondary or tertiary education, such as international matriculation examination or international higher education degree.

^b^Double qualification = high school and vocational school.

## Discussion

### Discussion of the results

In the current study, social and healthcare applicants’ ability to identify ethical situations was objectively assessed as part of the student selection process, which can be considered the first step to recruit future professionals. Based on the results of this study, the applicants’ performance in the ethics section varied. This suggests that all the students entering the education may not have the same basis for developing their ethical competence. This could be useful to acknowledge in social and healthcare education, both in theoretical studies and practical training. Ethical competence is part of the curricula around Europe, and the aim of education is to assist students in developing their ethical competence in theoretical and practical studies.^39,40^ Ethical situations are encountered daily, for example, in nursing, which requires the integration of both knowledge and practice in clinical learning environments.^1^ Furthermore, social and healthcare students enter practice learning environments from the very beginning of their studies (i.e. first or second semester of their studies). Practice supervisors (i.e. professionals in charge of supervising social and healthcare students in the practice learning environments) and managers at organisations (i.e. professionals in charge of supervising social and healthcare staff in hospital and other clinical settings) need to support (future) professionals in developing their ethical competence. According to previous studies,^41,42,43^ managers are responsible for facilitating the teams to act ethically. This requires managers to organise and allocate the work so that ethical reflections and discussion are encouraged and sufficient learning processes around the possible topics are carried out. Moreover, managers should offer ethics education, inform about ethical practices at workplaces, and support the teams to multidisciplinary ethics collaboration.^41–43^ We argue that managers and practice supervisors should be ethically competent and have moral courage themselves to be able to guide the community and future professionals as social and healthcare students enter practice learning environments.

The results also indicated that the applicants’ mean scores were rather low (below the centre of the positive scoring range) and that the number of applicants who failed the ethics section of the exam (i.e. did not reach the minimum pass score limit and thus were not considered in the final decision to offer a study place) can be considered rather high (22.7%). There may be three possible explanations for this. The first may be that the ethics section was quite difficult for applicants because of the difficulty level of the questions, which is partly supported by the results of the negatively skewed score distributions. The difficulty level of the questions should be further evaluated, for example, by using item response theory methods. The second explanation for the rather low scores may be the applicants’ exposure to the ethics section for the first time. Some examples of the exam questions and their types should be provided to applicants in the future. The third explanation is the use of dichotomous (true/false) response options and penalty scores (i.e. incorrect responses yield minus scores). In the future, it is important to deliberate on the use of dichotomous response options and penalty scores. Furthermore, to the best of our knowledge, this was the first attempt to assess objectively applicants’ ability to identify ethical situations in an entrance examination. Therefore, unfortunately, the results of this study cannot be compared or contrasted to previous studies. Previous studies about ethical competence of nursing applicants mainly focus on VBR which is criticised of lacking objectivity and discriminatory power especially in rejecting applicants.^27–29^

Based on the results of the current study, both applicants’ success (total score) and failed exam results in the ethics section can be explained by age, previous education, and place of birth (own, parent). These results are similar to previous studies, where statistically significant relationships between test-takers’ scores and background variables, such as age and previous education, have been reported.^44–46^ In entrance examinations, the fairness of the selection method is crucial. Therefore, it is important to analyse the possible effect of background variables and thoroughly consider why some applicants may score better than others. Our results showing a relationship between age and success in the ethics section could be explained by longer experience in work life and life experience in general and a possible previous higher education degree by age. Interestingly, the results indicated that high school graduates and applicants with higher education degrees scored better than those applicants with vocational diplomas. This result was not expected because most of applicants with vocational diplomas were most likely practical nurses (vocational qualification, not a higher education degree). However, this result can be somewhat positive, indicating that the ethics section does not require any previous professional knowledge from social and healthcare. On the other hand, the result may indicate that applicants with vocational diplomas do not necessarily have as good a theoretical basis as high school graduates. Furthermore, the result possibly indicates that the education of practical nurses does not necessarily prepare these professionals well enough to identify ethical situation, which is the basis of building ethical competence and needed in working life. It may also be possible that even practicing and experienced practical nurses lack ethical competence and should be further supported in their work by their managers. Future research should scope this issue. Studies on healthcare professionals’ ethical competence do exist, but the samples seem to include nurses in general and do not focus on practical nurses.^47^ The results concerning the place of birth can be discussed from several perspectives. It is possible that this result is associated with general learning skills and previous academic achievement. In a recent national follow-up study,^48^ pupils who had parents born outside Finland had a lower grade point average in their basic education certificate than those who had parents born in Finland. However, it should be noted that the results may also indicate that the ethics section should be further developed so that the language and cultural context are better considered. Overall, these results should be acknowledged in social and healthcare education and in working life, since it is possible that (future) professionals with different backgrounds may differ in their ethical competence and how it develops.

In this study, we objectively assessed social and healthcare applicants’ ability to identify ethical situations, which is part of ethical awareness.^16,20^ However, ethical awareness does not only involve the identification of the ethical situations but also the recognition of the consequences/implications and awareness of professionals’ roles and responsibilities in the situation.^18–22^ The ethics section of the UAS Exam is a newly developed entrance examination test, so further development is needed. In the future, critical analysis is needed to consider what other elements of ethical awareness and thus parts of ethical competence can be measured objectively using an electronic format in the student selection stage. Overall, objective assessment of ethical competence has been scarce − the few previously identified instruments measuring ethical competence reveal the fact that ethical competence is mainly measured subjectively.^47^ Because ethical competence is a multidimensional and hierarchical ability, we aimed to objectively assess the applicants’ ability to identify ethical situations. The use of structured assessment methods is also supported by the literature.^10^

### Limitations

The present study has some limitations that should be acknowledged. The sample size can be considered large. However, in analysis of the background variables, the number of applicants in different comparison groups varied. For example, the number of the youngest applicants (<20 years) and those born outside Finland/parent(s) born outside Finland were small, which may have affected the validity of these results. Furthermore, in the future, more descriptive and detailed background information could be collected for further analysis. The results also indicate that even though the ethics section is valid, it also seems to be quite difficult. Therefore, further development and psychometric testing of the ethics section is needed.

## Conclusion

Ethics section was used to objectively assess social and healthcare applicants’ ability to identify ethical situations. According to the results, applicants’ success in the ethics section varies and thus their ability to identify ethical situations differs. This indicates that all the students entering the education may not have the same basis for developing their ethical competence. This needs to be recognised especially during first practical placements. Students may need support in identifying ethical situations in practice and in further developing their ethical competence. Looking at the results, applicants from different backgrounds may benefit from further development of the inclusiveness of the ethics section. Furthermore, future research with longitudinal design should focus on the assessment of ethical competence and how it develops during education and to what extent may the ethics section scores in student selection phase be predictive of study success in theoretical and clinical studies. Finally, further development of the ethics section is needed, especially, the difficulty level of the exam section should be further analysed to make decisions on the question types used in the future.

## Data Availability

The authors do not wish to share the data due to privacy/ethical restrictions.
